# Medical Therapies Beyond Infliximab for Perianal Fistulizing Crohn’s Disease: Current Evidence and Future Perspectives

**DOI:** 10.3390/jcm15187327

**Published:** 2026-09-21

**Authors:** Esteban Fuentes-Valenzuela, Francisco Javier Tarrasó, Belén Botella, Pilar Nos, Isabel Vera, Marisa Iborra

**Affiliations:** 1 IBD Unit, Gastroenterology and Hepatology Department, Hospital Universitario Puerta de Hierro, 28222 Majadahonda, Spain; 2 Inflammatory Bowel Disease Research Group, Health Research Institute La Fe (IIS La Fe), IBD Unit, Gastroenterology Department, La Fe University and Polytechnic Hospital, 46026 Valencia, Spain

**Keywords:** Crohn’s disease, perianal fistulizing Crohn’s disease, advanced therapies

## Abstract

Perianal fistulizing Crohn’s disease (pfCD) represents one of the most challenging manifestations. Optimal management requires a multidisciplinary approach combining surgical drainage, appropriate imaging, and advanced medical therapies. While infliximab remains the first-line medical therapy, primary non-response or secondary loss of response occurs in many patients, which highlights the need for effective alternative treatments. This narrative review summarizes the current evidence regarding medical therapies beyond infliximab, including biologics, small molecules, and emerging therapeutic strategies. A literature search of PubMed, Embase, and Scopus was conducted up to February 2026 to identify studies evaluating medical treatments of pfCD. Current evidence suggests that ustekinumab and upadacitinib remain effective following anti-TNF failure, while vedolizumab demonstrates limited efficacy. Evidence supporting IL-23 inhibitors, including risankizumab and guselkumab, is limited but rapidly evolving, with ongoing prospective trials expected to clarify their role. Novel approaches such as advanced dual-targeted therapy, subcutaneous infliximab optimization, hyperbaric oxygen therapy, and exclusive enteral nutrition have also shown encouraging preliminary results in selected patients. However, most available data derive from post hoc analyses, retrospective cohorts, or small prospective studies, with considerable heterogeneity in outcome definitions and limited incorporation of standardized radiological endpoints. Future adequately powered randomized controlled trials using harmonized clinical and magnetic resonance imaging -based outcomes are essential to establish optimal treatment sequencing and improve long-term fistula healing. Until then, individualized multidisciplinary management remains fundamental to optimizing outcomes in patients with pfCD.

## 1. Introduction

Perianal fistulizing Crohn’s disease (pfCD) represents one of the most disabling and therapeutically challenging phenotypes. It encompasses a broad spectrum of manifestations, including fistulas, abscesses, anal ulcers, skin tags, anal stenosis, and, less frequently, anal carcinoma [1]. The burden of perianal disease is substantial: the cumulative risk of developing perianal disease after a diagnosis of CD has been estimated at 26.2%, and population-based cohorts indicate that up to two-thirds of affected patients require at least one surgical procedure during the course of their disease [2]. Beyond its anatomical and surgical complexity, perianal CD profoundly impairs quality of life. It affects physical functioning, sexual health, body image, and psychological well-being, with depressive symptoms reported in up to 73% of patients and suicidal ideation in approximately 13% [3].

Unlike luminal CD, pfCD is characterized by distinctive pathogenic mechanisms that extend beyond chronic intestinal inflammation. Current evidence suggests that fistula formation and persistence result from a complex interplay of genetic susceptibility, microbial dysbiosis, dysregulated innate and adaptive immune responses, and defective tissue repair [4]. Persistent activation of innate and adaptive immune pathways resultstreatment goals have increasingly evolved in sustained production of pro-inflammatory cytokines, including TNF-α, IL-12, IL-23, IL-17, IL-22, IL-13, and transforming growth factor-β (TGF-β), which drive epithelial-to-mesenchymal transition (EMT), extracellular matrix remodeling, impaired tissue repair, and the maintenance of chronic fistula tract. Matrix metalloproteinases further contribute to tissue destruction and compromised wound healing, while alterations in the local microbiome appear to sustain chronic inflammation. These biological mechanisms provide the rationale for current and emerging therapeutic targets, including TNF inhibition, blockade of the IL-12/23 and IL-23 pathways, inhibition of leukocyte trafficking, Janus kinase (JAK) inhibition, and regenerative therapies aimed at promoting tissue repair. Moreover, the unique biology of pfCD may explain why therapies highly effective for luminal inflammation often achieve only partial success in achieving durable fistula healing [4].

Given this complexity, the management of pfCD requires a multidisciplinary approach involving gastroenterologists, colorectal surgeons, radiologists, specialist nurses, and, when appropriate, stoma and wound-care teams. Adequate control of local sepsis through examination under anesthesia, abscess drainage, and seton placement remains the cornerstone of successful medical treatment [5]. Increasingly, treatment goals are evolving. This data position allows us to move beyond simple cessation of fistula drainage toward a treat-to-target strategy incorporating radiological healing, sphincter preservation, avoidance of permanent stoma, and improvement in patient-reported outcomes.

Current therapeutic strategies combine early surgical management with advanced medical therapy. Anti-tumor necrosis factor (anti-TNF) agents, particularly infliximab, remain the cornerstone of first-line biological treatment for pfCD and are supported by the highest level of evidence [6,7]. However, primary non-response, secondary loss of response, incomplete fistula healing, treatment discontinuation, and the limited long-term durability remain major unmet needs. During the last decade, an improved understanding of the immunopathogenesis of pfCD has expanded the therapeutic landscape beyond TNF blockade to include anti-integrin therapy, IL-12/23 and selective IL-23 inhibitors, Janus kinase inhibitors, optimized anti-TNF strategies, mesenchymal stem cell therapy, and other emerging regenerative approaches [1,4]. However, the optimal positioning and sequencing of these therapies remain uncertain, as comparative evidence is scarce and dedicated randomized trials in pfCD are still limited. In this narrative review, the main aim was to critically appraise the current evidence supporting medical therapies beyond infliximab for pfCD. Rather than providing a simple overview of available drugs, we discuss the biological rationale underlying each therapeutic strategy, analyze the strengths and limitations of the available evidence, and propose a practical treatment algorithm to guide therapeutic sequencing in contemporary clinical practice.

## 2. Materials and Methods

We conducted a narrative review aiming to summarize the current evidence regarding medical therapies for pfCD. An initial literature search of PubMed, Embase, and Scopus was performed up to 23 February 2026 and was subsequently updated in August 2026 using the same databases and search concepts. The search strategy included combinations of the terms “perianal Crohn’s disease” and the names of the principal therapies evaluated in pfCD, including infliximab, subcutaneous infliximab, adalimumab, ustekinumab, vedolizumab, upadacitinib, filgotinib, guselkumab, mirikizumab, risankizumab, and topical therapies.

Eligibility was based on the relevance of the publication to the medical management of pfCD and the availability of clinically relevant efficacy and/or safety data. We prioritized fully published, peer-reviewed manuscripts, including randomized controlled trials, prospective studies, and relevant retrospective multicenter studies. Only full-text publications in English were considered. Conference abstracts and other publications without a complete peer-reviewed manuscript were excluded in order to maintain a homogeneous evidence base. Studies addressing exclusively surgical management or surgical outcomes, including seton placement, were excluded because surgical management was outside the scope of this review. Therefore, the discussion of surgical outcomes, although important within multidisciplinary management, was not assessed in this review.

Studies were selected according to their relevance to the predefined scope of the review, with greater emphasis placed on prospective and multicenter studies and on studies using clinically meaningful fistula outcomes. Because this was a narrative review, study selection was based on clinical relevance and methodological characteristics rather than a formal systematic review screening process.

The identified evidence was narratively synthesized and critically interpreted according to study design, sample size, prospective or retrospective nature, endpoint definitions, duration of follow-up, and the presence of clinical and/or radiological outcomes. No formal risk-of-bias or methodological quality assessment was performed, as the objective of this narrative review was to provide a clinically oriented synthesis rather than a systematic assessment of the certainty of evidence. This limitation was taken into consideration when interpreting comparative efficacy and therapeutic positioning.

## 3. Results

### 3.1. New Classification of Fistulizing Perianal Crohn’s Disease and Its Clinical Implications

Historically, the management of pfCD has relied on anatomical classifications, the most widely used being the Parks classification, which primarily describes fistula anatomy but provides limited guidance for therapeutic decision-making. A systematic review by Geldof et al. [8] identified 18 different classification systems, highlighting the lack of a universally accepted framework and the considerable heterogeneity in the assessment of pfCD. This variability has hampered both clinical decision-making and the comparison of outcomes across clinical studies.

To overcome these limitations, the international TOpCLASS Consortium proposed a novel dynamic classification system that integrates anatomical complexity with disease activity, reparability, patient-reported outcomes, and therapeutic goals. TOpCLASS recognizes that pfCD is a dynamic disease in which treatment objectives evolve and should be individualized according to both clinician- and patient-defined priorities [8].

The consortium established standardized MRI definitions for treatment response and healing. Radiological healing is defined as the absence of T2-weighted hyperintensity, complete fibrosis of the fistula tract, and, when gadolinium is administered, the absence of post-contrast enhancement on T1-weighted images. In contrast, radiological improvement requires at least one objective sign of response, including increasing fibrosis of the tract or a clear reduction in T2 hyperintensity, fistula diameter or length, abscess size, or contrast enhancement. These consensus definitions provide standardized imaging endpoints for both clinical practice and future therapeutic trials. Although MRI activity scores are currently recommended mainly for research purposes, the consensus also proposes practical reassessment intervals at 6 and/or 12 months after surgery or initiation of advanced medical therapy [9,10,11].

**Figure 1:**
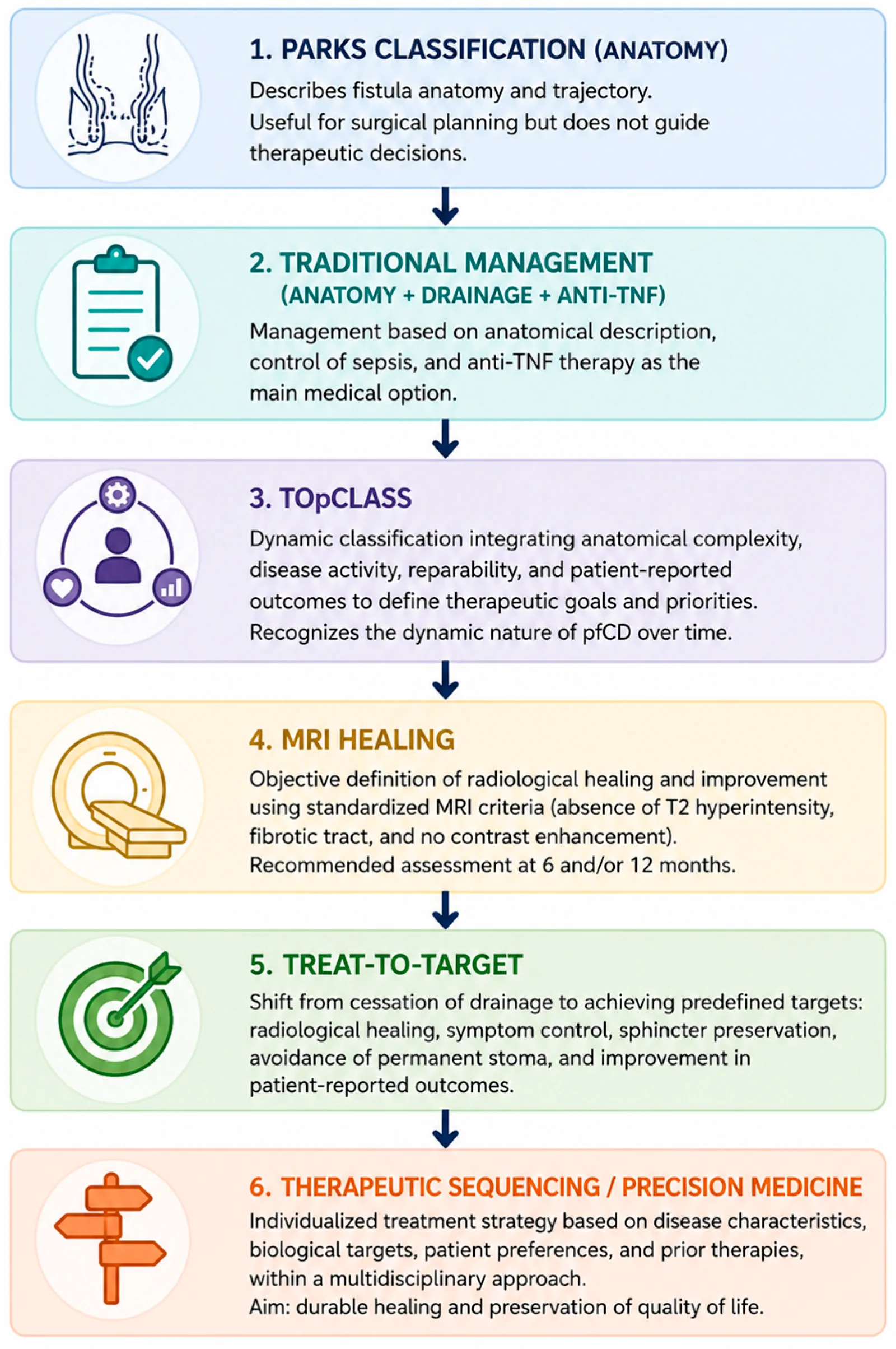
Evolution of disease classification and therapeutic targets in fistulizing perianal Crohn’s disease.

Taken together, these advances represent an important shift in the management of pfCD. Rather than focusing solely on cessation of fistula drainage, current strategies are increasingly moving toward a treat-to-target approach in which radiological healing, preservation of anorectal function, and patient-reported outcomes are key therapeutic objectives. This evolving framework has important implications for clinical trial design, treatment sequencing, and routine multidisciplinary care. The evolution from anatomical classification toward dynamic, MRI-based treat-to-target assessment and therapeutic sequencing is summarized in Figure 1.

### 3.2. Medical Treatment of Perianal Fistulizing Crohn’s Disease

The main prospective studies evaluating medical therapies for pfCD are summarized in Table 1. Importantly, the efficacy outcomes reported across studies are not directly interchangeable. Clinical fistula closure, cessation of fistula drainage, clinical response, and clinical remission reflect different aspects of disease activity. Similarly, combined clinical and MRI-based endpoints incorporate information beyond clinical assessment alone. Therefore, the outcomes presented in Table 1 should be interpreted according to the definitions and assessment methods used in each individual study, and comparisons should be made cautiously.

#### 3.2.1. Anti-TNF Therapies

Anti-TNF agents remain the best-established first-line biological therapy for pfCD. The pivotal randomized, double-blind clinical trial by Present et al. was instrumental in establishing the efficacy of infliximab in this setting, demonstrating complete fistula closure in more than 50% of treated patients compared with placebo [12]. Subsequently, the ACCENT II trial confirmed higher short-term fistula closure rates with infliximab compared with placebo at week 14 (46% vs. 13%); however, a substantial proportion of patients experienced relapse during long-term follow-up, highlighting the limited durability of response [13].

Adalimumab has also shown efficacy in pfCD. In the CHARM trial, an open-label induction therapy with adalimumab 80 mg was followed by 40 mg at week 2. At week 4, patients were randomized in a double-blind fashion to receive the placebo, adalimumab 40 mg eow, or adalimumab 40 mg weekly through week 56. The proportion of randomized responders achieving remission was significantly higher in the adalimumab 40 mg every other week and 40 mg weekly groups compared to the placebo group at both week 26 and week 56. Specifically, at week 26, the remission rates were 40%, 47%, and 17% (*p* < 0.001), while at week 56, the rates were 36%, 41%, and 12%, respectively (*p* < 0.001) [14]. Later, the ADAFI trial, a double-blind placebo-controlled trial, demonstrated the efficacy of combination therapy with adalimumab and ciprofloxacin. After adalimumab induction therapy, patients received adalimumab 40 mg eow with ciprofloxacin 500 mg or placebo twice daily for 12 weeks, with a follow-up until 24 weeks. The combined treatment resulted in higher clinical response rates at week 12 (71%) compared with adalimumab monotherapy (47%; *p* = 0.009) and higher CDAI change and mean IBDQ change at week 12; however, this advantage was not sustained at week 24, underscoring the transient benefit of adjunctive antibiotic therapy [15].

Evidence for certolizumab pegol is considerably more limited. In a post hoc analysis of the PRECISE study, complete fistula closure at week 26 was achieved in 36% of patients receiving certolizumab compared with 17% receiving placebo [16].

Given the frequency of secondary loss of response, therapeutic drug monitoring (TDM) may play an important role in optimizing anti-TNF therapy in pfCD. Higher infliximab trough levels have been associated with improved fistula healing, and the 2024 *Grupo Español de trabajo de Crohn y Colitis ulcerosa* (GETECCU) Position Statement recommends targeting infliximab concentrations of 20–25 μg/mL at week 2 and 10–15 μg/mL at week 6 during induction in patients with active pfCD, reflecting the higher inflammatory burden of this phenotype [25]. Reactive TDM may therefore be considered before concluding that anti-TNF therapy has failed, particularly in patients with secondary loss of response or incomplete fistula healing. However, evidence supporting proactive TDM in this setting remains limited. Subcutaneous infliximab has emerged as an attractive strategy for optimizing drug exposure because it provides more stable serum concentrations than intravenous administration. Although evidence in pfCD remains limited, a post hoc analysis of the REMSWITCH study included 40 patients with previous pfCD; however, only three had active perianal disease at the time of switching, limiting conclusions about fistula healing [26]. A recent French multicenter cohort of 183 patients reported clinical remission in 44.6% of patients with active disease at 6 months, while relapse-free survival among patients with inactive disease reached 94.3% and 87.9% at 6 and 12 months, respectively [27]. Furthermore, in a multicenter ENEIDA study, maintenance infliximab trough concentrations associated with sustained remission (approximately 12–13 μg/mL) were similar in patients with pfCD and luminal CD, suggesting that once remission has been achieved, patients with perianal disease may not require higher maintenance infliximab concentrations solely because of perianal involvement. Although fistula healing was not specifically evaluated, these findings may support the use of proactive TDM and provide additional pharmacokinetic rationale for subcutaneous infliximab in long-term maintenance [28].

Taken together, these studies firmly establish anti-TNF agents, particularly infliximab, as the cornerstone of medical therapy for pfCD. Nevertheless, durable fistula healing remains elusive for many patients, emphasizing the need for treatment optimization through TDM and pharmacokinetic strategies, as well as the development of effective therapeutic alternatives beyond TNF blockade.

#### 3.2.2. Vedolizumab

Evidence supporting vedolizumab in pfCD derives mainly from post hoc analyses and relatively small prospective cohorts. In the GEMINI 2 post hoc analysis, which evaluated 461 patients initiating vedolizumab maintenance therapy, 12% had at least one actively draining fistula at baseline. Fistula closure was achieved in 28% of vedolizumab-treated patients compared with 11% of those receiving placebo at week 14, with this difference persisting through week 52 (31% vs. 11%). Furthermore, patients treated with vedolizumab had a faster time to fistula closure [17].

The phase IV ENTERPRISE trial further supported the efficacy of vedolizumab, with 53.6% of patients achieving at least a 50% reduction in draining fistulas at week 30. However, the study was prematurely terminated because of slow recruitment and failed to demonstrate any benefit from an intensified induction regimen [18].

Real-world data have been less encouraging. In the prospective *Groupe d’Étude Thérapeutique des Affections Inflammatoires du Tube Digestif* (GETAID) cohort, vedolizumab achieved clinical success in only 22.5% of patients and radiological response in 38.4% at week 30. Moreover, recurrence occurred in nearly one-third of patients with inactive disease, and almost one-quarter required treatment escalation [19].

Taken together, these findings suggest that vedolizumab has modest efficacy in pfCD. While it remains a reasonable option in selected patients, particularly those with contraindications to systemic immunosuppression or a high infectious risk, its relatively slow onset of action and limited fistula healing rates make it a less attractive choice for patients with active fistulizing disease when more effective alternatives are available.

#### 3.2.3. Ustekinumab

Evidence supporting ustekinumab in pfCD has increased substantially over recent years. The first randomized placebo-controlled trial evaluated intravenous ustekinumab (6 mg/kg) followed by subcutaneous maintenance (90 mg every 8 weeks). Although the study was prematurely terminated because of slow recruitment, ustekinumab achieved a significantly higher rate of combined clinical and radiological remission at week 12 than placebo (62% vs. 25%; OR 5.1, 95% CI 1.07–24.4). Differences in secondary endpoints did not reach statistical significance [20]. This study provides the most important evidence for ustekinumab, but its findings should be interpreted cautiously. The study was prematurely discontinued because of slow recruitment. Although the observed week-12 combined remission rate was higher with ustekinumab than placebo, the very small sample size probably limited the differences in the rest of the outcomes. Furthermore, the randomized phase was relatively short, and no head-to-head comparison with other advanced therapies was performed.

The GETAID BioLAP multicenter retrospective study including 148 patients reported a clinical success rate of 38.5% and radiological response in 50% of cases [29]. Additional retrospective cohorts, although not specifically designed to assess pfCD outcomes and often limited by small sample sizes, have consistently reported clinical response rates ranging from 40% to 60% [30,31,32,33].

Notably, a recent real-world retrospective analysis of 108 patients—43.5% of whom had complex perianal fistulas—reported clinical remission and response rates of 40.7% and 63%, respectively, along with significant improvements in the Perianal Crohn’s Disease Activity Index and quality-of-life measures. Radiological healing was observed in 44.8% [34]. On the other hand, whether ustekinumab trough levels could predict remission in pfCD was assessed in a retrospective study including a total of 89 patients, where the receiver operating characteristic curve (ROC) identified concentrations ≥3.95 µg/mL at 16/20 weeks as predictive of clinical fistula remission (AUC: 0.791; sensitivity: 73.8%; specificity: 78.6%) [35].

Finally, a systematic review and meta-analysis of 9 studies included a total of 396 patients with active pfCD. The pooled proportions of patients experiencing fistula response were 41.0%, 39.7% and 55.9% at weeks 8, 24, and 52, respectively. Regarding fistula remission, the pooled proportions were 17.1%, 17.7%, and 16.7% at weeks 8, 24, and 52, respectively [36].

These data support ustekinumab as one of the better-supported biologic options after anti-TNF failure in pfCD, although the available evidence remains limited and heterogeneous. Importantly, evidence supporting efficacy does not necessarily establish superiority or preferential positioning over other advanced therapies, as adequately powered head-to-head comparative trials in pfCD are lacking.

#### 3.2.4. Small Molecules

Small molecules, particularly Janus kinase (JAK) inhibitors, have emerged as promising therapeutic options for pfCD owing to their rapid onset of action, oral administration, lack of immunogenicity, and broad anti-inflammatory activity. Their biological rationale is further supported by the involvement of the JAK–STAT pathway in the pathogenesis of perianal Crohn’s disease.

The phase II DIVERGENCE 2 trial provided the first prospective evidence supporting this therapeutic class. In this randomized, placebo-controlled study, 57 patients with active pfCD received filgotinib 200 mg, filgotinib 100 mg, or placebo for 24 weeks. The primary endpoint was a combined clinical and radiological fistula response, defined as the closure of at least one draining external opening without pelvic MRI evidence of fluid collections >1 cm. Combined fistula response was achieved in 47.1% of patients receiving filgotinib 200 mg compared with 29.2% in the 100 mg group and 25% in the placebo group. Although serious adverse events were numerically more frequent in the 200 mg group, the study provided the first proof-of-concept that JAK inhibition may be effective in pfCD [21]. These encouraging findings paved the way for the evaluation of more selective JAK1 inhibitors.

Upadacitinib, a selective JAK1 inhibitor, has demonstrated encouraging results in this setting and currently represents the small molecule with the strongest evidence in pfCD. A post hoc analysis of the phase III U-EXCEL and U-EXCEED trials evaluated 143 patients with fistulizing disease, most of whom had perianal fistulas. Resolution of perianal fistula drainage at the end of induction occurred in 44.7% of patients treated with upadacitinib 45 mg compared with 5.6% in the placebo group. During maintenance therapy, 28.6% of patients receiving upadacitinib 15 mg maintained fistula closure, whereas no such responses were observed in the placebo arm. External fistula opening closure was also achieved more frequently with upadacitinib. Nevertheless, these analyses lacked objective imaging endpoints, limiting conclusions regarding true fistula healing [22].

These findings have recently been supported by real-world studies. In a multicenter North American cohort involving ten tertiary referral centers, clinical response and remission were achieved in 45.5% and 39.4% of patients, respectively. Importantly, MRI follow-up was available in approximately 60% of the cohort, demonstrating radiological improvement in 55.1% and complete radiological healing in 11.5% of patients [37]. Similarly, the French GETAID cohort reported sustained clinical remission in 26% of patients at both 6 and 12 months, supporting the durability of response in routine clinical practice [38]. Additional observational studies in refractory CD populations have consistently confirmed favorable effectiveness and safety, although perianal-specific outcomes were often reported only as secondary analyses [39,40,41]. Interestingly, clinical benefit has also been described in patients with TOpCLASS 4 disease after proctectomy, suggesting that JAK inhibition may retain activity even in highly refractory pfCD [42]. However, despite these encouraging findings, objective radiological evidence remains limited and further prospective studies are needed to establish the extent and durability of fistula healing with upadacitinib.

Taken together, current evidence positions upadacitinib as one of the most promising therapeutic options beyond anti-TNF therapy for pfCD. Its rapid onset of action, oral administration, and encouraging efficacy in both clinical trials and real-world cohorts make it an attractive alternative following anti-TNF failure. However, safety considerations are an important component of upadacitinib positioning. As with other JAK inhibitors, treatment is associated with an increased risk of infections, particularly herpes zoster, and attention should be paid to the potential risks of venous thromboembolism, major adverse cardiovascular events, and malignancy [43]. Accordingly, although upadacitinib may represent an attractive small-molecule option because of its efficacy and oral administration, its use should be individualized according to age, comorbidities, cardiovascular and thromboembolic risk, and infection risk.

#### 3.2.5. Guselkumab, Risankizumab and Mirikizumab

Selective IL-23 inhibition represents one of the most promising emerging therapeutic strategies for pfCD, given the central role of the IL-23/Th17 axis in chronic inflammation, epithelial-to-mesenchymal transition, and impaired tissue repair. However, unlike ustekinumab, clinical evidence specifically addressing pfCD remains scarce.

The largest available real-world study evaluated risankizumab in a multicenter cohort of 520 patients, of whom 29% had a history of pfCD. Active perianal disease decreased from 8.9% at baseline to 5.2% in week 52. Among the 15 patients with actively draining fistulas at treatment initiation, drainage cessation was achieved in 40% at week 12 and 50% at week 26 [44]. Although these findings are encouraging, the number of patients with active fistulizing disease was small, precluding a definitive conclusion [44].

More robust evidence is expected from the ongoing FUZION-CD trial, the first phase III randomized, placebo-controlled study specifically designed to evaluate guselkumab in patients with active pfCD. Importantly, the study incorporates objective MRI endpoints and prolonged follow-up, which should provide valuable information regarding both clinical and radiological healing [45].

At present, no dedicated data are available for mirikizumab in pfCD. Although patients with pfCD were included in the VIVID program, perianal-specific outcomes have not been reported [46,47].

The current evidence regarding IL-23 in pfCD is limited, primarily based on a small number of patients with pfCD in larger retrospective studies. Therefore, these results should be interpreted with caution, as no dedicated retrospective or prospective study has evaluated the outcomes for pfCD. However, this situation is expected to evolve soon, with promising insights anticipated from the Fuzion-CD trial. Despite the limited clinical evidence, selective IL-23 blockade is supported by a strong biological rationale and has demonstrated remarkable efficacy in luminal CD. Therefore, it is likely to become an increasingly important therapeutic option for pfCD as dedicated clinical data emerge.

#### 3.2.6. Advanced Dual Therapies

A French single-center prospective study evaluated the efficacy of advanced dual therapy for pfCD. The primary outcome was clinical effectiveness. A total of 33 patients were included, and the most common combination was infliximab with ustekinumab in 75.8%. After a median of 27.4 months, 48.5% and 97.0% of patients were in complete clinical remission and in response, respectively. Complete radiological remission was achieved in 24.2% of patients. The treatment was well tolerated, and grade 1 and 2 adverse events were reported in 15.2% [23].

However, the use of dual biologic or small-molecule therapy has been recommended only for highly refractory, carefully selected patients treated at specialized centers. Thus, in a systematic review, including 288 dual-therapy regimens, this risk for adverse events and serious adverse events occurred in 31% and 6.5% of cases, respectively. Therefore, the safety issue should be carefully considered before initiating dual therapies [48].

#### 3.2.7. Comparison Studies

Head-to-head randomized controlled trials comparing advanced therapies for fistulizing pfCD are currently lacking. Consequently, treatment positioning relies largely on retrospective comparative studies, propensity score-matched analyses, and indirect evidence. Although these studies should be interpreted cautiously, they provide valuable insights into therapeutic sequencing in routine clinical practice.

The efficacy of infliximab and adalimumab as first-line therapy was compared in a retrospective cohort study from two large tertiary centers. The primary endpoint was the fistula response and remission at 6 and 12 months. At 6 months, clinical improvement was significantly higher in the infliximab group (64.9% versus 34.8%; *p* < 0.01) [49]. The comparison between adalimumab and infliximab was summarized in a recent systematic review and meta-analysis, including a total of 6 observational studies, five of them of retrospective design, and a total of 590 patients. The healing rate was comparable for both groups, risk ratio (RR) 0.90, 95% confidence interval (CI) 0.76–1.06, *p* = 0.20. No differences were observed for clinical assessment or radiological assessment by separate analyses [50].

Thus, Shani et al. [51] performed a multicenter international retrospective cohort study including patients with pfCD who failed a first anti-TNF therapy, with propensity score matching. The primary outcome was the clinical response in active pfCD. A total of 486 patients were included. In the second-line group, 79.5% of patients treated with ustekinumab (UST) achieved a clinical perianal response, compared to 58.9% with vedolizumab (OR 4.47, 95% CI, 1.94–10.28, *p* < 0.001) and 48.7% with anti-TNFs (OR 5.29, 95% CI, 2.39–11.71, *p* < 0.001). For third-line therapies, 77.6% of ustekinumab treated patients achieved clinical perianal response, compared to 59.2% with vedolizumab (OR 9.96, 95% CI, 2.6–38.4, *p* < 0.001) and 55.1% with anti-TNFs (OR 12.03, 95% CI, 2.99–48.47, *p* < 0.001).

Furthermore, Casanova et al. [52], in a Spanish multicenter retrospective cohort study, compared the treatment persistence, clinical remission, and safety of ustekinumab and vedolizumab for pfCD. The remission rate for ustekinumab was 54% and 46% for vedolizumab, with a similar rate of adverse events. Moreover, Eldesouki et al. [53] performed a retrospective propensity-matched cohort study, comparing the effectiveness of ustekinumab and vedolizumab using the data from the TriNetX US collaborative Network. The main outcome was the perianal-specific clinical events, including definitive fistula surgery, incision and drainage, diversion procedures, and antibiotic use (metronidazole or ciprofloxacin), assessed at 1 and 2 years after biological initiation. In this study, ustekinumab was associated with a lower risk of perianal fistula surgery (HR: 0.66, 95% CI: 0.50–0.89; *p* < 0.01) and composite surgical events (HR: 0.70, 95% CI: 0.54–0.87; *p* < 0.01) at 1 year. Importantly, at two years this benefit persisted as well, with lower use of antibiotics (HR: 0.86, 95% CI: 0.75–0.93; *p* = 0.04).

A monocentric Chinese retrospective cohort study compared the efficacy of infliximab versus ustekinumab as first-line therapy, and the primary endpoint was the proportion of patients achieving fistula remission. A total of 16.3% of patients receiving ustekinumab achieved fistula remission, and 22.9% in the infliximab group (*p* = 0.419) [54].

Although comparative evidence remains entirely observational, the available data consistently favors mechanism switching over anti-TNF cycling after infliximab failure. Among currently available biologics, ustekinumab has shown the most consistent effectiveness across comparative cohorts, whereas vedolizumab appears less effective for active fistulizing disease. Whether JAK inhibitors or selective IL-23 inhibitors will modify this therapeutic hierarchy remains one of the key unanswered questions for future prospective studies.

Table 2 shows the evidence-informed treatment framework for medical management of perianal fistulizing Crohn’s disease. Importantly, this framework should not be interpreted as a definitive therapeutic hierarchy. In the absence of head-to-head randomized controlled trials specifically evaluating pfCD, differences in efficacy across therapies cannot be reliably established through indirect comparisons of heterogeneous studies. Infliximab remains the therapy with the strongest supporting evidence, whereas the relative positioning of other biologics and small molecules after anti-TNF failure remains uncertain and should be individualized according to clinical context and patient characteristics. Current evidence suggests that the therapeutic paradigm of pfCD is progressively shifting from empirical treatment selection toward mechanism-based therapeutic sequencing, integrating pharmacokinetic optimization, disease biology, and objective radiological targets.

#### 3.2.8. Adjunctive Therapies

(A) Hyperbaric oxygen therapy

Hyperbaric oxygen therapy (HBOT) has emerged as a potential adjunctive treatment for refractory pfCD. The prospective HOT-TOPIC study evaluated 20 patients with therapy-refractory pfCD treated with HBOT and followed for 60 weeks. Significant improvements were observed in both the Perianal Disease Activity Index and the modified Van Assche MRI score at weeks 16 and 60, suggesting sustained clinical and radiological benefit [49]. However, the study lacked a control group and included a limited number of highly selected patients [24].

Real-world evidence remains scarce. However, real-world evidence is limited. Thus, a recent case series of 6 patients who underwent this therapy for pfCD, including two patients who underwent total proctocolectomy with ileal-anal pouch anastomosis and four who had fecal diversion with ileostomy or colostomy, and were on advanced therapies except one patient. Patients received between 10 and 40 sessions, and four patients reported symptomatic relief, although on physical examination and imaging, improvement was only noted in one patient [55].

Overall, although HBOT appears to be a safe adjunctive therapy with encouraging preliminary results, current evidence is insufficient to support its routine use. At present, it should be considered only in highly selected patients with refractory disease and within experienced multidisciplinary centers.

(B) Thiopurines and tacrolimus

Before the biological era, thiopurines and calcineurin inhibitors were explored as therapeutic options for pfCD, although their role is currently limited. In a prospective open-label study, azathioprine combined with antibiotics achieved higher response rates than antibiotics alone (48% vs. 15% at week 20), suggesting a modest benefit as adjunctive therapy rather than as standalone treatment [56].

Tacrolimus has also been evaluated in pfCD. In a randomized placebo-controlled trial, oral tacrolimus improved fistula drainage more frequently than placebo (43% vs. 8%); however, complete fistula remission remained uncommon and adverse events were more frequent [57]. Likewise, an exploratory trial of topical tacrolimus failed to demonstrate benefit in fistulizing disease, although some improvement was observed in ulcerating perianal lesions [58].

Overall, current evidence does not support the routine use of thiopurines or tacrolimus for pfCD in the era of biologics and small molecules. Their role is now largely restricted to selected refractory cases or as adjunctive therapies in highly individualized clinical scenarios.

(C) Exclusive Enteral nutrition

A recent Chinese retrospective cohort study evaluated the radiological and clinical outcomes of exclusive enteral nutrition (EEN) in pfCD. The primary endpoint was radiological healing after receiving EEN for 8–12 weeks. In this study, a total of 12.5% of patients reported radiological healing, while 77.6% reported radiological improvement. Clinical fistula response and remission were achieved in 55.9% and 47.4% of patients, respectively [59]. This is the largest published study evaluating EEN for pfCD, as previously only case reports and small observational studies [60,61,62] existed.

Therefore, this very limited evidence for EEN supports that it could be an option in very selected patients. The evidence for other specific diets or combination of diet and advanced therapies for pfCD is currently lacking.

## 4. Sequencing Advanced Medical Therapies in Perianal Fistulizing Crohn’s Disease

TDM has become a cornerstone of anti-TNF optimization in pfCD. Higher infliximab trough concentrations during induction have consistently been associated with improved clinical and radiological fistula outcomes, and the 2024 GETECCU Position Statement recommends target trough concentrations of 20–25 μg/mL at week 2 and 10–15 μg/mL at week 6 in patients with active pfCD [25]. However, the optimal maintenance trough concentration required for durable fistula healing remains uncertain. Emerging evidence suggests that the pharmacokinetic targets required to maintain remission may differ from those needed to induce fistula healing. Therefore, before declaring anti-TNF failure and considering a change in therapeutic mechanism, inadequate drug exposure and immunogenicity should be excluded whenever possible through proactive TDM [63,64,65,66].

Infliximab remains the recommended first-line biologic for pfCD according to current ECCO, ACG, and BSG guidelines [6,66,67,68]. In patients with an inadequate response, objective reassessment with pelvic MRI should be performed to distinguish persistent inflammatory fistulas from predominantly fibrotic tracts before therapeutic sequencing is considered [10].

Beyond anti-TNF therapy, no international guideline establishes a clear therapeutic hierarchy because comparative randomized trials are lacking. ECCO recommends adalimumab or upadacitinib following infliximab failure [6], whereas the ACG and BSG guidelines also include ustekinumab and vedolizumab among acceptable alternatives [66,67]. Consequently, treatment selection currently relies on indirect evidence, real-world comparative studies, patient characteristics, and physician experience.

Recent comparative studies suggest that mechanism switching may be preferable to anti-TNF cycling in patients with mechanistic treatment failure despite adequate infliximab exposure. Among currently available biologics, ustekinumab has demonstrated the most consistent comparative evidence, showing superior clinical outcomes to vedolizumab and anti-TNF recycling in propensity score-matched cohorts, together with favorable radiological and long-term real-world results [51,52,53]. Upadacitinib has emerged as another highly promising option because of its rapid onset of action, oral administration, and encouraging efficacy in both phase III trials and real-world cohorts, although dedicated comparative and long-term radiological studies are still lacking [22,37,38,39,40,41,42]. In contrast, vedolizumab appears to provide more modest efficacy in active pfCD and may be preferentially reserved for patients in whom safety considerations outweigh the need for rapid fistula healing [17,18,19].

Selective IL-23 inhibitors represent one of the most promising future therapeutic strategies given their strong biological rationale, although dedicated evidence in pfCD remains limited and their precise position within the treatment algorithm awaits the results of ongoing prospective trials [44,45,46]. It may offer a more favorable safety profile than JAK inhibitors or combination therapies. Thus, in a systematic review with meta-analysis of six RCTs including 3640 patients treated with IL-23 inhibitors, the incidence of any adverse events was comparable with placebo, with no increase in serious infections [69]. Therefore, it could be considered for patients at higher risk of infections, such as older individuals or those with multiple comorbidities.

**Figure 2:**
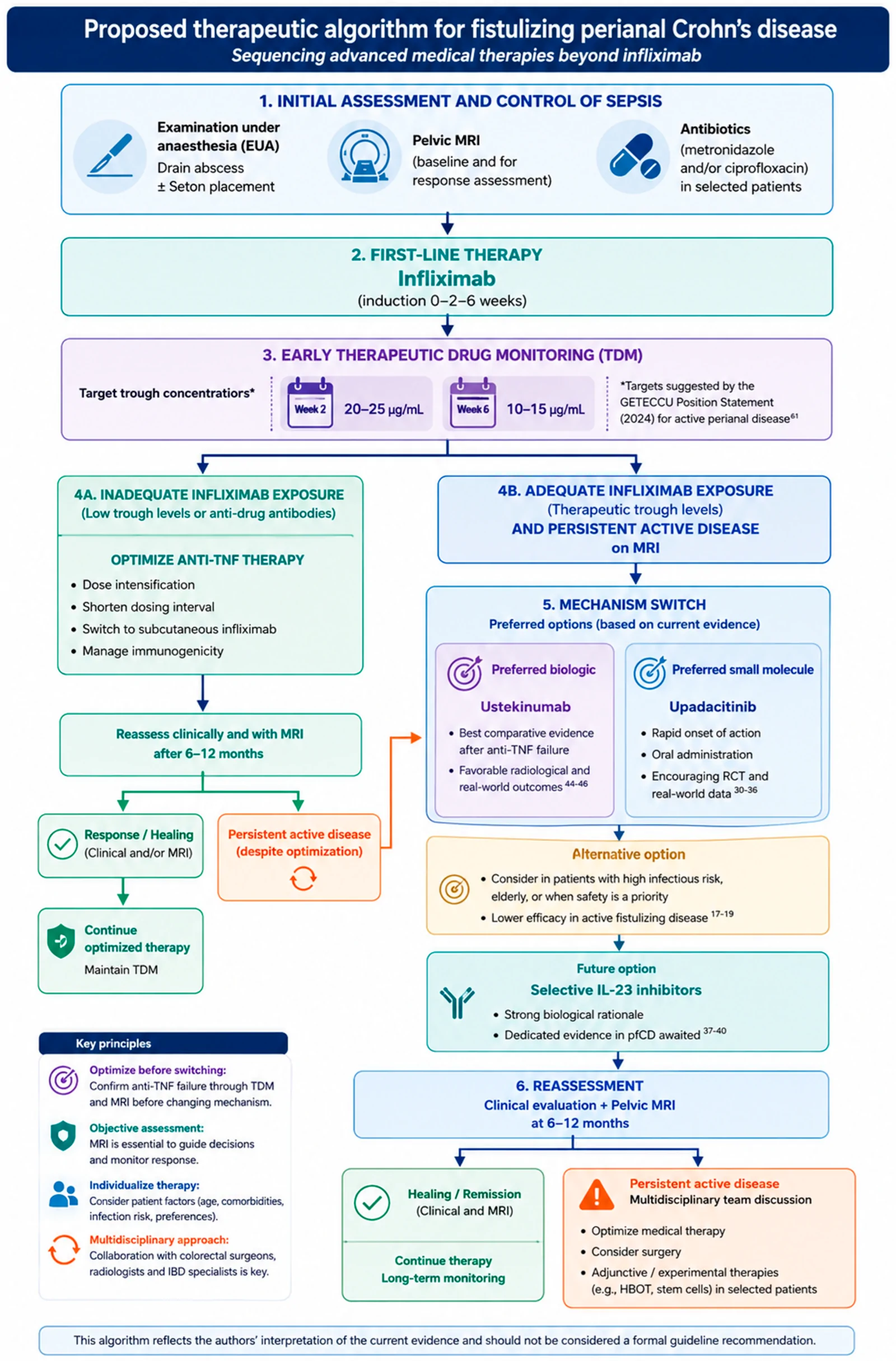
Proposed therapeutic algorithm for fistulizing perianal Crohn’s disease based on currently available evidence. The algorithm integrates early control of sepsis, optimization of anti-TNF therapy through therapeutic drug monitoring, mechanism switching after pharmacokinetic failure has been excluded, and objective radiological reassessment to guide subsequent treatment decisions. This proposal reflects the authors’ interpretation of the available evidence and should not be considered a formal guideline recommendation [21,22,23,25,26,27,34,35,36,37,38,39,40,41,42,46,47].

Based on the currently available evidence, therapeutic sequencing in pfCD should no longer rely solely on the chronological availability of drugs, but rather on objective assessment of disease activity, pharmacokinetic optimization, and the mechanism underlying treatment failure. The overall evidence supporting the currently available advanced therapies is summarized in Table 3. Based on these data, we propose a practical treatment algorithm integrating early control of sepsis, pharmacokinetic optimization, objective radiological reassessment, and mechanism-based treatment selection. The proposed therapeutic algorithm is shown in Figure 2.

## 5. Current Limitations and Future Directions

Despite the growing number of publications evaluating medical therapies for pfCD, high-quality evidence remains remarkably scarce. Only a limited number of randomized controlled trials have been specifically designed for pfCD, whereas most prospective data derive from post hoc analyses of pivotal luminal Crohn’s disease trials or small observational cohorts. Furthermore, objective radiological assessment has been inconsistently incorporated, despite MRI being considered the gold standard for evaluating treatment response and fistula healing.

An additional limitation is the substantial heterogeneity in outcome definitions across published studies. Clinical response, clinical remission, fistula healing, radiological response, and radiological healing have all been defined differently, with considerable variation in the timing of assessments. This heterogeneity complicates comparisons between studies and limits the feasibility and interpretability of meta-analyses [70,71].

To address these limitations, an international multidisciplinary expert panel recently proposed standardized recommendations for the design of randomized clinical trials in pfCD [72]. The recommendations call for standardized eligibility criteria, combined clinical and MRI-based remission endpoints, and consistent evaluation windows for induction and maintenance outcomes.

Future clinical trials should move beyond simple assessment of fistula drainage and incorporate objective MRI healing, patient-reported outcomes, therapeutic drug monitoring, and long-term durability of remission. Head-to-head comparative trials evaluating therapeutic sequencing after anti-TNF failure, together with studies integrating radiological treat-to-target strategies, represent the most important unmet research priorities in pfCD.

In conclusion, the management of pfCD requires a multidisciplinary approach integrating medical, surgical, and radiological expertise. Anti-TNF therapy, particularly infliximab, remains the cornerstone of first-line treatment. However, long-term fistula healing remains challenging because of frequent loss of response and limited durability. Emerging therapies, including ustekinumab, upadacitinib, and selective IL-23 inhibitors, are expanding the therapeutic landscape, although high-quality comparative evidence remains limited. Recent advances in MRI-based assessment, therapeutic drug monitoring, and the understanding of disease biology support a shift from empirical treatment selection toward mechanism-based therapeutic sequencing. Future studies should focus on defining the optimal sequencing of advanced therapies using standardized clinical and radiological endpoints, allowing a more personalized approach to the management of pfCD.

## Figures and Tables

**Table 1:** Main prospective studies evaluating the efficacy of medical therapies for perianal fistulizing Crohn’s disease.

Author	Drug	Study Design	Sample Size (Patients)	Clinical Outcome	Radiological Outcome	Year
Present et al. [12].	Infliximab	Randomized controlled trial	94	Complete fistula closure: 55% (5 mg/kg), 38% (10 mg/kg) vs. 13% (placebo)	no	1999
Sands et al. [13].	Infliximab	Randomized controlled trial (ACCENT II)	282	Absence of draining fistulas at week 54: 36% vs. 19% (placebo)	no	2004
Colombel et al. [14].	Adalimumab	Randomized controlled trial (CHARM)	117	Complete fistula closure: 36% vs. 13% (placebo)	no	2007
Dewint et al. [15].	Adalimumab + ciprofloxacin	Randomized controlled trial (ADAFI)	76	Week 12: clinical response 71% vs. 47%; Week 24: no significant difference	no	2014
Schreiber et al. [16].	Certolizumab	Post hoc analysis (PRECISE)	58	Fistula closure: 36% vs. 17% (*p* = 0.038)	no	2011
Feagan et al. [17].	Vedolizumab	Post hoc analysis (GEMINI 2)	57	Fistula closure: week 14, 28% vs. 11%; week 52, 31% vs. 11% (placebo)	no	2018
Schwartz et al. [18].	Vedolizumab	Randomized controlled trial (ENTERPRISE)	32	≥50% reduction in draining fistulas at week 30: 53.6%	Decrease in 1.1 points in the Van Assche score	2022
Chapuis-Biron et al. [19].	Vedolizumab	Prospective multicenter cohort	151	Clinical success 22.5%	Radiological response 38.4%	2020
Wils et al. [20].	Ustekinumab	Randomized placebo-controlled trial (USTAP)	32	Combined clinical/radiological remission: 62% versus 25%	yes	2026
Reinisch et al. [21].	Filgotinib (200/100 mg/placebo)	Randomized controlled trial (Phase II)	57	Combined clinical and radiological fistula response at week 24: 47.1%/29.2%/25%	yes	2024
Colombel et al. [22].	Upadacitinib	Post hoc analysis (U-EXCEL and U-EXCEED)	128	Drainage resolution 44.7% vs. 5.6%; external opening closure 22%	no	2024
Fathallah et al. [23].	Advanced Dual therapy	Prospective cohort study	33	Clinical improvement/remission: 48.5%/97.0%	Complete radiological remission: 24.2%	2025
Lansdorp et al. [24].	Hyperbaric oxygen therapy	Prospective cohort study	20	Week 16: PDAI decreased from 7.5 to 4	Modified Van Assche index decreased from 9.2 to 7.3	2022

**Table 2:** Evidence-informed treatment framework for medical management of perianal fistulizing Crohn’s disease.

Clinical Scenario	Preferred Strategy	Rationale
**Anti-TNF naïve active pfCD**	Infliximab + surgical control of sepsis	Highest-quality evidence (RCTs)
**Inadequate infliximab levels or immunogenicity**	Dose optimization ± proactive TDM/SC infliximab	Avoid premature mechanism switch
**Primary anti-TNF failure or mechanistic failure**	Mechanism switch	Cycling anti-TNF appears less effective
**Well-supported biologic option after anti-TNF failure**	Ustekinumab	Most consistent comparative real-world evidence
**Well-supported small molecule after anti-TNF failure**	Upadacitinib	Rapid onset; encouraging clinical and MRI data
**Selected patients with high infectious risk/elderly**	Vedolizumab	Excellent safety despite lower efficacy
**Future option**	Selective IL-23 inhibitors	Strong biological rationale; ongoing RCTs

**Table 3:** Evidence supporting advanced therapies for fistulizing perianal Crohn’s disease.

Therapy	Dedicated pfCD Trial	MRI Assessment	Comparative Data	Current Evidence
**Infliximab**	Yes	Limited	Available	Established
**Vedolizumab**	Yes	Limited	Limited	Modest
**Ustekinumab**	Yes (USTAP)	Yes	Available	Promising
**Upadacitinib**	Post hoc	Limited	Emerging	Promising
**IL-23 inhibitors**	Ongoing	Yes	Not available	Emerging

## Data Availability

No new data were created or analyzed in this manuscript. Data sharing is therefore not applicable.

## Abbreviations

The following abbreviations are used in this manuscript: AGA, American Gastroenterological Association; AUC, area under the curve; BSG, British Society of Gastroenterology; CD, Crohn’s disease; CDAI, Crohn’s Disease Activity Index; CI, confidence interval; ECCO, European Crohn’s and Colitis Organisation; EEN, exclusive enteral nutrition; EMT, epithelial-to-mesenchymal transition; GETAID, Groupe d’Étude Thérapeutique des Affections Inflammatoires du Tube Digestif; HBOT, hyperbaric oxygen therapy; HR, hazard ratio; IBD, inflammatory bowel disease; IBDQ, Inflammatory Bowel Disease Questionnaire; IFX, infliximab; IL, interleukin; JAK, Janus kinase; JAK–STAT, Janus kinase–signal transducer and activator of transcription; MRI, magnetic resonance imaging; OR, odds ratio; PDAI, Perianal Disease Activity Index; pfCD, perianal fistulizing Crohn’s disease; RCT, randomized controlled trial; ROC, receiver operating characteristic; RR, risk ratio; SC, subcutaneous; TDM, therapeutic drug monitoring; TNF, tumor necrosis factor; TOpCLASS, TOpCLASS classification.
